# Doxycycline induces apoptosis via ER stress selectively to cells with a cancer stem cell-like properties: importance of stem cell plasticity

**DOI:** 10.1038/s41389-017-0009-3

**Published:** 2017-11-29

**Authors:** Takashi Matsumoto, Takeshi Uchiumi, Keisuke Monji, Mikako Yagi, Daiki Setoyama, Rie Amamoto, Yuichi Matsushima, Masaki Shiota, Masatoshi Eto, Dongchon Kang

**Affiliations:** 1 Department of Clinical Chemistry and Laboratory Medicine, Kyushu University, 3-1-1, Maidashi, Higashi-ku, Fukuoka, 812-8582 Japan; 2 Department of Urology, Graduate School of Medical Sciences, Kyushu University, 3-1-1, Maidashi, Higashi-ku, Fukuoka, 812-8582 Japan; 3 Department of Nutritional Sciences, Faculty of Health and Welfare, Seinan Jo Gakuin University, 1-3-5 Ibori, Kokurakita-ku, Kitakyushu, 803-0835 Japan

## Abstract

Tumor heterogeneity can be traced back to a small subset of cancer stem cells (CSCs), which can be derived from a single stem cell and show chemoresistance. Recent studies showed that CSCs are sensitive to mitochondrial targeting antibiotics such as doxycycline. However, little is known about how cancer cells undergo sphere formation and how antibiotics inhibit CSC proliferation. Here we show that under sphere-forming assay conditions, prostate cancer cells acquired CSC-like properties: promoted mitochondrial respiratory chain activity, expression of characteristic CSC markers and resistance to anticancer agents. Furthermore, those CSC-like properties could reversibly change depending on the culture conditions, suggesting some kinds of CSCs have plasticity in tumor microenvironments. The sphere-forming cells (i.e. cancer stem-like cells) showed increased contact between mitochondria and mitochondrial associated-endoplasmic reticulum (ER) membranes (MAM). Mitochondrial targeting doxycycline induced activating transcription factor 4 (ATF4) mediated expression of ER stress response and led to p53-upregulated modulator of apoptosis (PUMA)-dependent apoptosis only in the cancer stem-like cells. We also found that doxycycline effectively suppressed the sphere formation in vitro and blocked CD44v9-expressing tumor growth in vivo. In summary, these data provide new molecular findings that monolayer cancer cells acquire CSC-like properties in a reversible manner. These findings provide important insights into CSC biology and a potential new treatment of targeting mitochondria dependency.

## Introduction

The subpopulation of tumor cells commonly known as tumor-initiating cells, or cancer stem cells (CSCs), plays a critical role in tumorigenesis and is present in various types of cancer^1^. CSCs are characterized by extensive abilities in self-renewal and differentiation as unidirectional cellular hierarchies and may contribute to tumor progression, recurrence and metastasis^2–8^. These characteristics suggest that CSCs could be a critical target for cancer therapy.

The sphere-forming assay is commonly used to culture stem-like cancer cell lines on a low adhesion plate in a serum-free medium^8, 9^. In these conditions, approximately one percent of prostate cancer (DU145) monolayer cells are able to form sphere-forming colonies and this small population was thought to be CSCs in this assay. However, little is known as to how sphere-forming cells are selected or generated under those conditions from cultured monolayer cell and why these sphere-forming cells can represent CSCs.

The stemness is maintained in special environments called microenvironments or niche^10, 11^. Such stem or progenitor cells generally were irreversibly differentiated, that is, it was, thought that once differentiated cells did not return to stem cells. Recently it has been widely accepted that malignant tumors are heterogeneous and contain CSCs^12–14^. From a point of view of the general concept on stemness, CSCs may be also maintained irreversibly in tumor microenvironments^15^. However, the  definition of CSCs is not clearly settled yet^8, 10^. Instead, several characteristic properties are raised: expression of particular genes^16^, chemo/radio-resistance^17, 18^, regeneration of tumors from a single cell^19^ and so on. In this article, we called the CD44v9 positive cells with the CSC properties as cancer stem-like cells (CSC-like) because we did not examine a stemness of these cells.

Mitochondria is responsible for the production of adenosine triphosphate by oxidative phosphorylation (OXPHOS) and also play a important roles in Ca^2+^ buffering, β-oxidation, reactive oxygen species (ROS) production and apoptosis^20^. The Warburg effect states that tumor cells are defective in mitochondrial OXPHOS, then tumor cells depend on high levels of aerobic glycolysis for a ATP source to promote cellular growth^21^. However, recent studies have suggested that the mitochondrial OXPHOS are required for tumor growth and metastasis^22^. Another report also demonstrated that CSCs showed increased mitochondrial oxygen consumption rate (OCR)^23^.

We previously reported that oncogenic HRas indirectly suppressed the mitochondrial OCR, however, oxygen consumption is essential for tumorigenesis^24^. Serum depletion induced CSC-like properties, which argued by an increase in CSC markers expression and chemo-resistance. Thus, we speculated that cancer cells adapt under the tumor microenvironment and acquire the CSC-like properties.

Recent studies showed that antibiotics, which suppress mitochondrial translation and function, were cytotoxic activity against mammalian cancer cells especially CSCs^25–27^, but the mechanism of antibiotics inhibition on cell growth remains unclear. In mammalian cells, the endoplasmic reticulum (ER) and mitochondria show a tight junction and interaction with each other, which showed as the mitochondria-associated ER membrane (MAM)^28–31^. Mitochondria translation inhibition by the antibiotics, which induce protein imbalance derived from mitochondria and nuclear, and mitochondrial dysfunction^32, 33^, can also trigger activating transcription factor 4 (ATF4)-mediated integrated stress response genes expression^34, 35^. However, the mechanisms linking MAM integrity and ER stress during sphere formation remain poorly understood.

Cancer cells avoid apoptosis through anti-apoptotic proteins upregulation and/or pro-apoptotic proteins downregulation^36^. p53-upregulated modulator of apoptosis (PUMA) is a BH3-only protein and acts as a key mediator of cytosolic pro-apoptotic p53 function. For example, PUMA is strongly induced by ER stress and may involve in ER stress-induced apoptosis in many human cancer cells^37^.

In this study, we examined the sphere-forming process of prostate cancer cell lines and CSC-like properties such as stem cell marker gene expression and mitochondrial function. Next, we investigated whether CSC-like properties were unidirectional. Then, we examined how antibiotics which target mitochondrial translation inhibition suppress CSC proliferation in vitro and whether doxycycline induces the ER stress response and apoptosis in sphere-forming cells. In a mouse xenograft model, we investigated the CD44v9 expression under both monolayer and sphere-forming culture conditions and whether doxycycline suppressed CD44v9-expressing tumor growth in vivo.

## Results

### CSC-like properties of sphere-forming PC-3 cells

Sphere-forming tumor cells are believed to contain CSCs subpopulation that might play a crucial role in chemoresistance^8, 38, 39^. Sphere-forming assays are an in vitro technique to assay clonogenic growth potential of both normal and neoplastic cells^8^. We thus first investigated the characteristics of CSCs which derived from sphere-forming assays in various cell lines. PC-3 prostate cancer cells and *RasG12V* transformed mouse embryonic fibroblast (MEF) could proliferate in sphere-forming conditions, however RWPE-1 normal prostate cells or non-transformed MEFs did not (Supplementary Fig. S1a). These results suggest that carcinogenesis is important for a sphere-forming ability.

**Fig. 1:**
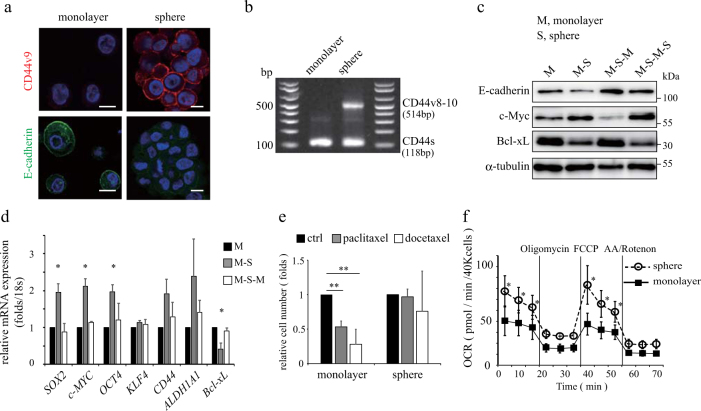
Sphere-forming PC-3 cells shows the CSC-like properties with plasticity **a** Immunofluorescence staining of CD44v9 and E-cadherin in monolayer and sphere-forming PC-3 cells. Scale bar = 10 μm. **b** CD44v8-10 and CD44s mRNA expression in monolayer and sphere-forming cells were performed by RT-PCR. **c** Immunoblotting analysis of E-cadherin, c-MYC, Bcl-xL and β-tubulin protein in the change of monolayer cells (3 days) to sphere-forming cells (3 days). M indicates monolayer, S indicates sphere-forming. **d** Relative mRNA expression of CSC marker in the change of monolayer (3 days) to sphere forming cells (3 days). Data were normalized to the expression level in monolayer for each RNA species. M indicates monolayer cells, S indicates sphere-forming cells. Data shows the mean ± SD of triplicates. *p < 0.05. **e** Relative cell number using MTS assay for monolayer and sphere-forming cells treated with 100 nM paclitaxel and 10 nM docetaxel for 3 days. Data were normalized to the absorbance level in monolayer for each species. Data shows the mean ± SD of triplicates. **p < 0.01. **f** OCR was measured using the Seahorse XF24 analyzer for monolayer and sphere-forming cell. Oligomycin, carbonylcyanide p-trifluoromethoxyphenylhydrazone (FCCP), rotenone, and antimycin A (AA) were added at the same time point for each experiment. Data shows the mean ± SD of quadruplicates. *p < 0.05

We observed the extracellular expression of CD44v9, which is one of the several markers associated with CSCs, in sphere-forming PC-3 cells but not in monolayer culture cells (Fig. 1a). We also confirmed the CD44 splicing variant (CD44v8-10) expression  in sphere forming cells by RT-PCR (Fig. 1b and Supplemental Fig. S1b) and a higher expression level of CD44v9 and oncogenic c-MYC proteins by western blotting in the sphere-forming cells than the monolayer cells (Fig. 1c and Supplementary Fig. S1c). We also found the increased expression of a variety of CSC markers such as *SOX2*, *c-MYC*, *OCT4*, *CD44*, *CD44v8-10* and *ALDH1A1* in sphere-forming PC-3 cells (Fig. 1d). In addition, we observed that the N-cadherin and Snail overexpression and E-cadherin reduction in sphere-forming cells. That is, the cells may indicate epithelial to mesenchymal transition (Supplemental Figs. S1c, d).

Because CSCs are relatively resistant to cancer chemotherapeutic agents, we next examined their chemotherapy resistance. Paclitaxel and docetaxel, which are commonly used for prostate cancer therapy, inhibited monolayer PC-3 cell growth (Fig. 1e). However, these anti-proliferative effects were not observed in sphere-forming cells, suggesting that sphere-forming cells are resistant to chemotherapy. Thus sphere-forming PC-3 cells harbor many properties typically observed in CSCs. On the other hand, their stemness, i.e., an ability of self-renewal and differentiation, are not examined yet. In this regard, we consider sphere-forming PC-3 cells as CSC-like at this stage.

The mitochondrial OXPHOS are necessary for tumor initiation, proliferation and metastasis^22^. Osteosarcoma 143B Rho^0^ cells lacking mitochondrial DNA (mtDNA) do not show a sphere-forming ability, suggesting that the mitochondrial OXPHOS is essential for sphere formation (Supplementary Fig. S1e). We next measured the OCR using a Seahorse Flux Analyzer. As shown in Fig. 1f, sphere-forming PC-3 cells showed an approximate 50% increase in mitochondrial respiration including basal respiratory capacity, OCR-linked maximal respiratory capacity and the ATP turnover value compared with monolayer cells. Sphere-forming PC-3 cells showed an increase in mitochondria membrane potential (MMP) and a decrease in ROS production (Supplementary Figs. S1f and g).

Next, we measured the mtDNA, mtRNA and several respiratory complex proteins encoded by mtDNA or the nuclear genome. Q-PCR analysis in sphere-forming PC-3 cells revealed no increase of mtDNA or mtRNA compared with monolayer cells (Supplementary Figs. S2a, b), suggesting that the mitochondrial alterations induced by sphere-forming were not due to alterations of mitochondrial genetic materials. We observed an increase in the COX1 and COX3 proteins encoded by mtDNA in the sphere-forming cells compared to the monolayer cells (Supplementary Fig. S2c), suggesting that sphere formation affects mitochondrial translation and the increased respiration is due in part to increased levels of respiratory complex proteins. These results indicate that an increase in mitochondrial OCR function was observed during the sphere formation, suggest that an increase of mitochondrial OCR might be a target for chemotherapy.

Recent studies have suggested that the PI3K-AKT pathway is upregulated in sphere forming prostatic DU145 cells and high level activity of PI3K-AKT are important for the maintenance and the generation of DU145 stem-like cell populations^40^. We found that sphere-forming PC-3 cells displayed an increased activation of the PI3K-AKT pathway (Supplementary Fig. S2d).

### Sphere-formation cell show CSC-like properties with plasticity

Since sphere-forming cells exhibit the CSC-like phenotype, we investigated whether the expression of CSC markers would reversibly change between monolayer and sphere-forming cells. The expressions of CSC marker genes and proteins reversibly change between monolayer and sphere-forming cultures (Figs. 1c, d), suggesting that the cells reversibly lose and obtain the gene expression pattern characteristic of CSCs depending on the culture conditions. These results raised the possibility that a so -called CSC subset contains CSC-like cells exhibiting plasticity in tumor microenvironments.

### Doxycycline inhibits the proliferation of sphere-forming cells

We observed that sphere-forming PC-3 cells showed increased OXPHOS activity. Some kinds of antibiotics, such as doxycycline, have been previously shown to inhibit mitochondrial translation, because mitochondria evolved from bacteria that were initially phagocytosed by eukaryotic cells at first between 1 and 2 billion years ago. Thus, we next investigated whether antibiotics such as doxycycline and chloramphenicol could inhibit sphere-forming of cancer cells derived from several prostate and bladder cancer lines.

**Fig. 2:**
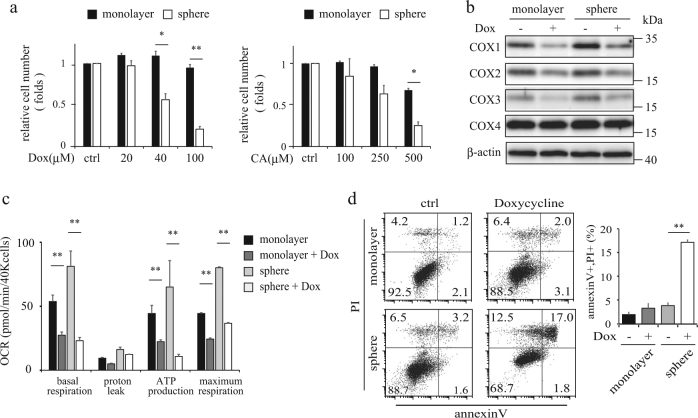
Doxycycline decreases OCR and induces the apoptosis in sphere-forming PC-3 cells **a** Relative cell number compared to no drug treatment by MTS assay in monolayer and sphere forming cells treated with various concentrations of doxycycline (Dox) and chloramphenicol (CA) for 3 days. Data shows the mean ± SD of triplicates. *p < 0.05, **p < 0.01. **b** Immunoblotting analysis of mtDNA-encoded COX1, COX2 and COX3, nuclear-encoded COX4 and β-actin protein in the monolayer and sphere-forming cells treated with 40 μM doxycycline for 48 h. **c** OCR was measured using the Seahorse XF24 Analyzer in monolayer and spheres with 40 μM doxycycline for 24 h. Data shows the mean ± SD of triplicates. **p < 0.01. **d** Flow cytometric analysis of apoptosis in monolayer and sphere-forming cells treated with 40 μM doxycycline for 24 h. In the graph on the right, the rates of the population of AnnexinV( + )/PI( + ) are indicated. A representative experiment out of three is shown. All data shows the mean ± SD of triplicates. **p < 0.01

Doxycycline inhibited sphere-forming of PC-3 cancer cells with an IC-50 of ~40 µM (Fig. 2a, Supplementary Fig. S3a). Similar results were also observed in bladder cancer KK47, KU-7 and T24 cells (Supplementary Fig. S3b). Chloramphenicol, another mitochondrial translation inhibitor, also inhibited sphere-forming of PC-3 cells with an IC-50 of ~250 µM (Fig. 2a). Interestingly, doxycycline and chloramphenicol did not affect the viability of various monolayer cancer cells (Fig. 2a and Supplementary Fig. S3b), suggesting that these antibiotics can be used to eradicate cells harboring gene expression patterns characteristic of CSCs.

Because those antibiotics inhibit mitochondrial translation, we investigated whether the antibiotics directly act on mitochondria to limit respiration. In both monolayer and sphere-forming cells, doxycycline and chloramphenicol decreased expression of COX1, COX2, and COX3 encoded by mtDNA, but not COX4 (a nuclear-encoded protein) (Fig. 2b and Supplementary Fig. S3c), and reduced mitochondrial respiration (Fig. 2c).

To investigate whether the anti-proliferative effects of doxycycline were due to cell death, we next examined whether doxycycline induced apoptosis in sphere-forming PC-3 cells. Doxycycline treated PC-3 cells induced apoptosis, as determined by cleaved caspase-3 expression (Supplementary Fig. S3d) and increased Annexin V-FITC and propidium iodide (PI) positive cells (Fig. 2d), while doxycycline did not affect the CD44 splicing variant expression in Sphere-forming cells (Supplemental Fig. S3e). These results suggested that antibiotics such as doxycycline are anti-proliferative mainly in sphere-forming cells but not monolayer cells, despite mitochondrial translational inhibition in both cellular states.

### MAM formation and FACL4 expression in sphere-forming cells

**Fig. 3:**
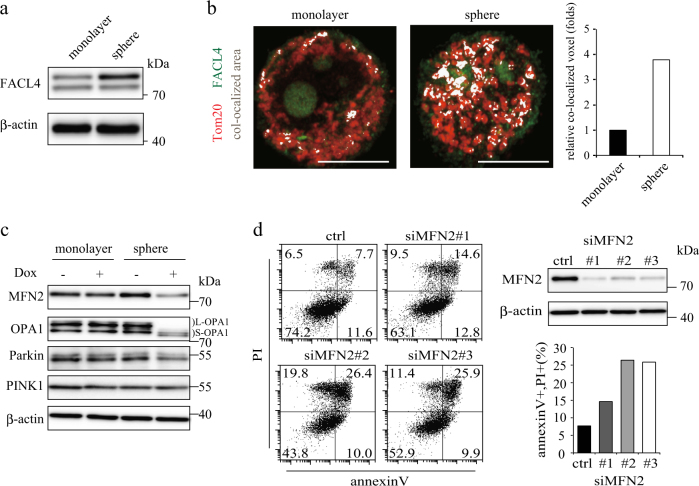
MAM formation promote doxycycline-induced apoptosis in sphere-forming cells **a** Immunoblotting analysis of FACL4 in monolayer and spheres. β-actin served as an internal loading control. **b** Representative immunostaining of FACL4 (Green) and TOM20 (Red) in monolayer and sphere forming cells. Colocalized image (white dot) was confirmed in three-dimensional reconstructions from the raw confocal image using IMARIS. Relative colocalized voxel normalized in monolayer was indicated in right panel. Scale bar = 10 μm. **c** Western blot analysis of MFN2, OPA1, Parkin, and PINK1 protein in monolayer and sphere-forming cells treated with 100 μM doxycycline for 24 h. β-actin served as an internal loading control. **d** Flow cytometric analysis of apoptosis in monolayer and sphere-forming cells with transfected with MFN2 siRNAs as indicated and treated with 40 μM doxycycline for 24 h. In the graph on the right, the rates of the population of AnnexinV(+)/PI(+) are indicated. Immunoblotting analysis of Mfn2 protein (on the right) indicates the efficacy of the siRNA

We next examined why apoptosis was only induced in sphere-forming PC-3 cells despite the inhibition of mitochondrial translation by doxycycline in both the cell cultures. We focused on MAM, which is a separate membrane compartment that connects the ER to mitochondria. Compared with monolayer cells, sphere-forming PC-3 cells showed higher levels of FACL4, a MAM marker protein (Fig. 3a), raising the possibility that sphere-forming cells modulate mitochondrial function by affecting MAM formation. The fluorescence of the MAM marker FACL4 around the rim of the mitochondria marked by TOM20 (Fig. 3b, highlighted in white in a colocalized area panel of fluorescence images) shows contact sites between mitochondria and MAM in sphere-forming PC-3 cells. The contact sites increased around 4-fold in the sphere-forming cells (Fig. 3b, a quantitation graph). To examine the morphology of mitochondria in sphere-forming PC-3 cells after doxycycline treatment, we stained cells with TOM20 and DAPI and examined by differential confocal microscopy. In monolayer PC-3 cells, mitochondria appeared to bind to each other in a filamentous network and were abundant throughout the cytoplasm (Supplemental Fig. S4a, upper left). In sphere-forming PC-3 cells, mitochondria appeared a rounded morphology (Supplemental Fig. S4a, lower left). After doxycycline treatment, mitochondria became more fragmented and scarce in the sphere-forming cells but not much changed in the monolayer cells (Supplemental Fig. S4a, right panels).

Mitochondrial network formation depends on the balance between enhanced function of the mitochondrial division mechanism and the inhibition of mitochondrial fusion proteins^41^. To investigate the mitochondrial division phenotype in doxycycline-treated sphere-forming cells, we examined the mitochondrial fission and fusion components. The expression of long OPA1 was reduced and short OPA1 was increased after doxycycline treatment in the sphere-forming cells (Fig. 3c), suggesting that mitochondrial fragmentation was due to reduced mitochondrial fusion activity.

In addition, MFN2, a MAM and mitochondria-localized protein that tethers the ER to mitochondria, was also reduced after doxycycline treatment in the sphere-forming cells (Fig. 3c). Mfn2 depletion in mouse fibroblasts resulted in the disruption of the ER-mitochondria contact sites, increased distance between ER mitochondria organelles and induction the ER and mitochondrial morphology change^42^. To investigate whether MFN2 is involved in doxycycline-induced apoptosis in sphere-forming cells, we performed FACS analysis after siRNA-mediated MFN2 knockdown. Knockdown of MFN2 resulted in increased apoptotic cells in the sphere-forming cells, suggesting that the reduced MFN2 expression is involved in doxycycline-induced apoptosis in the sphere-forming cells (Fig. 3d).

### Doxycycline induces ER stress response in sphere-forming cells

**Fig. 4:**
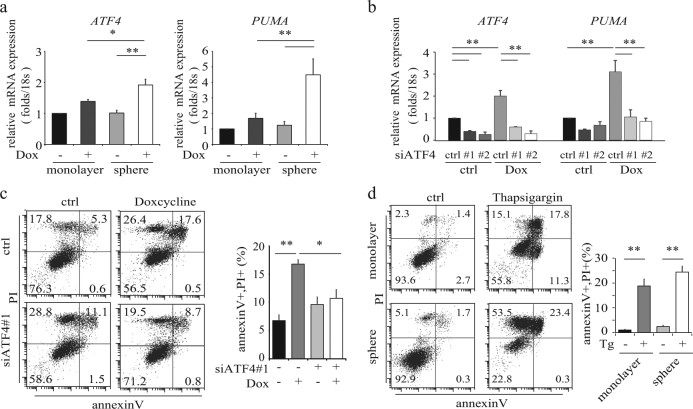
Doxycycline induces ER stress response and PUMA expression in sphere-forming cells **a** Relative *ATF4* and *PUMA* mRNA expression in monolayer and sphere forming cells treated with 40 μM doxycycline for 24 h. Data were normalized to the expression level in monolayer for each RNA species. Data shows the mean ± SD of triplicates. *p < 0.05, **p < 0.01. **b** Relative mRNA expression of *ATF4* and *PUMA* for spheres transfected with ATF4 (#1 and #2) or control siRNA. Data were normalized to the expression level in monolayer for each RNA species. **p < 0.01. **c** Flow cytometric analysis of apoptosis in sphere-forming cells transfected with ATF4#1 siRNA and treated with 40 μM doxycycline for 24 h. In the right panel, the rates of subpopulations of Annexin V ( + )/PI ( + ) are shown. *p < 0.05, **p < 0.01. **d** Flow cytometric analysis of apoptosis in monolayer and sphere-forming cells treated with 1 μM thapsigargin (Tg) for 24 h. In the right panel, the rates of subpopulation of Annexin V (+)/PI (+) are shown. **p < 0.01

Because MAM formation was modulated in the sphere-forming cells (Fig. 3b) and mitochondrial translation inhibition is involved in the ER stress response, we investigated whether doxycycline enhances the ER stress response in sphere-forming PC-3 cells to induce apoptosis. Doxycycline increased ATF4 expression in the nuclei more strongly in the sphere-forming cells (Fig. 4a and Supplementary Fig. S4b) and induced integrated gene response activation (*ATF3*, *DDIT3/CHOP*, *FGF21* and *GDF15*) similarly (Supplementary Fig. S4c). Doxycycline also increased the levels of pro-apoptotic factor *PUMA* (Fig. 4a) only in the sphere-forming cells but not in the monolayer cells. These results suggested that doxycycline induces the ER stress response in the sphere-forming cells.

To investigate whether ATF4 is involved in doxycycline-induced *PUMA* expression and apoptosis, we performed siRNA-mediated ATF4 knockdown in the sphere-forming cells. ATF4 knockdown attenuated doxycycline-mediated *PUMA* expression (Fig. 4b) and doxycycline-mediated apoptosis in sphere-forming PC-3 cells (Fig. 4c and Supplemental Fig. S4e). Knockdown of PUMA expression also attenuated doxycycline induced apoptosis (Supplementary Fig. S4f). These results suggested that doxycycline-induced ER stress activated apoptosis through *PUMA* expression in the sphere-forming cells, but not the monolayer cells.

To investigate whether ER stressors induce apoptosis in monolayer PC-3 cells, we treated monolayer- and sphere-forming PC-3 cells with thapsigargin, which inhibits ER Ca^2+^ ATPase. Thapsigargin-induced apoptosis in both monolayer and sphere-forming PC-3 cells (Fig. 4d and Supplemental Fig. S4g), suggesting that the defect of ER stress response by doxycycline is involved in apoptosis deficits in monolayer culture.

### Doxycycline induces ROS production

Because doxycycline induces ROS production and ROS is involved in the apoptotic pathway, we investigated ROS production in sphere-forming PC-3 cells. We observed increased ROS production after doxycycline treatment in the sphere-forming cells compared with the monolayer cells (Supplementary Fig. S5a). Pretreatment with *N*-acetyl-cysteine, which attenuates ROS amounts decreased doxycycline-induced apoptosis in the sphere-forming cells, suggesting that doxycycline-induced ROS production is partially involved in apoptosis (Supplementary Fig. S5b).

### Reduced expression of anti-apoptotic Bcl-xL in sphere-forming cells

To determine whether anti-apoptotic Bcl-2 family proteins were involved in doxycycline-induced apoptosis, we quantitatively analyzed PCR to confirm the expression levels of several anti-apoptotic and pro-apoptotic Bcl-2 family proteins (Supplementary Fig. S4d). The mRNA and protein expression of anti-apoptotic Bcl-xL was decreased in sphere-forming PC-3 cells (Supplementary Figs. S5c and d), suggesting that downregulation of anti-apoptotic Bcl-xL expression in the sphere-forming cells is also involved in doxycycline-induced apoptosis. The siRNA-mediated knockdown of Bcl-xl increased the doxycycline-induced apoptosis in the sphere-forming cells (Supplementary Fig. S5e).

### Doxycycline inhibits growth of CD44v9-expressing cell in a xenograft model

**Fig. 5:**
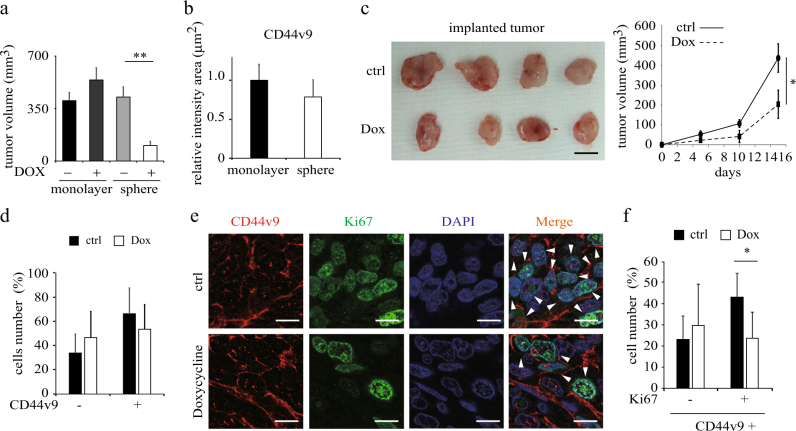
Doxycycline inhibits CD44v9 positive cell proliferation in the xenograft model **a** The monolayer or sphere-forming PC-3 cells with or without doxycycline (40 μM) pretreatment were injected into nude mice (Balb/c-nu) and the tumor volumes were measured at a 19 days. *n* = 3 for each group. **p < 0.01. **b** Relative intensity area of CD44v9 expression analyzed by Keyence software in the xenograft model implanted with monolayer or spheres. Data were normalized to the expression level in monolayer. **c** Changes in tumor volume in Balb/c-nu mice at 15 days after the xenograft. *n* = 4 for each group. The mice were treated with 60 mg/kg of doxycycline or saline by intraperitoneal administration every day after sphere implantation. In the right panel, the tumor volume indicates chronological change. *p < 0.05. **d** The % cell number of CD44v9 staining after doxycycline were estimated. **e** Immunofluorescence staining of CD44v9, Ki67 and DAPI in the xenograft model treated with saline or doxycycline for 15 days. Each arrow indicates the cells with Ki67 and CD44v9 co-staining. Scale bar = 10 μm. **f** The % cell number of CD44v9 and Ki67 co-staining are estimated. *p < 0.05

A previous study reported that sphere-forming prostate DU145 cells-initiated xenograft tumors showed larger tumor size compared than monolayer cells^9^. So, we next examined the effects of doxycycline on tumorigenesis using monolayer and sphere-forming PC-3 cells in xenografts in nude mice. To examine the tumor growth potential of PC-3 cells, monolayer or sphere-forming PC-3 cells with or without doxycycline pretreatment were injected into nude mice and the tumor volumes were counted. Interestingly, there were no significant differences in tumor volume among the monolayer and sphere-forming groups without doxycycline. However, doxycycline-pretreated sphere-forming PC-3 cells showed drastically reduced capacity of tumor growth (Fig. 5a and Supplementary Fig. S6a), suggesting that anti-proliferation effect of CSCs by doxycycline in vitro maintain in vivo.

In both the sphere cell and monolayer cell-derived xenograft tumors, CD44v9 was clearly heterogeneously expressed (Supplementary Fig. S6b) in contrast to that it is homogenously expressed in the cells within spheres and not expressed in the monolayer cells (Fig. 1a). Interestingly, the cell population expressing CD44v9 was the same in the sphere forming derived xenograft tumors as the monolayer cell derived ones (Fig. 5b). These results suggested that xenograft tumors derived from either spheres or monolayers adapt to the xenograft circumstances similarly.

We next investigated effects of doxycycline treatment of nude mice. Following doxycycline or normal saline intraperitoneal injection for 15 consecutive days, tumor samples were removed from mice. Tumor size was significantly suppressed in mice treated with doxycycline compared with saline treated (*p* < 0.05; Fig. 5c and Supplementary Fig. S6c). Ki67 is a marker for proliferating cells. The cell number of Ki67 positive or negative was not significantly changed irrespective of doxycycline treatment (Supplementary Fig. S6b). We showed that no difference in CD44v9 expression after Doxycycline treatment in mouse xenograft (Fig. 5d). However, Ki67 and CD44v9 double positive cells were significantly reduced after doxycycline treatment while CD44v9(+)/Ki67(-) cell number was not changed (Figs. 5e and f), suggesting that doxycycline affected CD44v9 expressing cell proliferation in the xenograft mouse model.

## Discussion

**Fig. 6:**
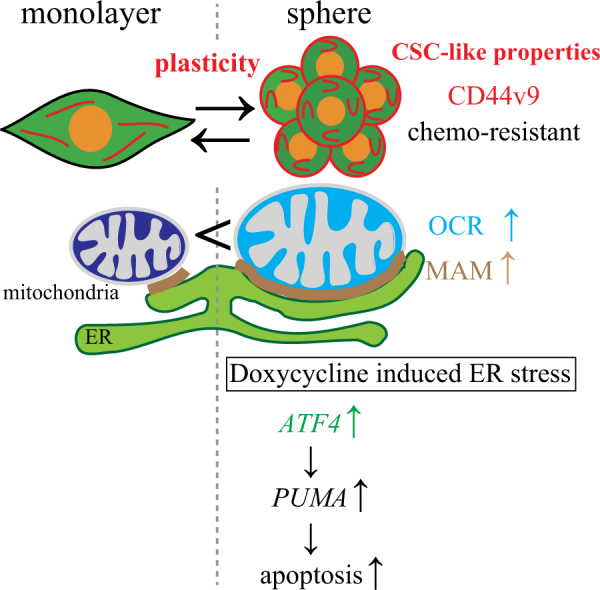
Scheme of CSC-like properties between monolayer and sphere formation and doxycycline induces apoptosis via ER stress (i) sphere-forming cultures of prostate cancer cells showed increased mitochondrial OCR, CD44v9 expression and resistance to anticancer agents; (ii) during sphere formation, cancer cells display CSC-like properties with plasticity; (iii) sphere-forming cancer cells show increased MAM formation; (iv) doxycycline disrupts MAM formation leading to ER stress response and apoptosis in sphere forming cells (CSC-like properties)

We demonstrated that doxycycline inhibited cancer stem-like cell proliferation in vitro and in vivo. The major new findings of this study are as follows (Fig. 6): (i) sphere-forming cultures of prostate cancer cells show increased mitochondrial respiratory chain activity, CSC marker expression and resistance to anticancer agents; (ii) during sphere formation, cancer cells display CSC-like properties with plasticity; (iii) sphere-forming cancer cells show increased MAM formation; (iv) doxycycline disrupts MAM formation, causes ER stress response and induces apoptosis; and (v) doxycycline inhibits growth of cancer cells that express CD44v9 in vivo.

Sphere-forming assay is considered an in vitro technique to observe a clonogenic growth potential of both normal and neoplastic cells and assessment of the self-renewal and differentiation potential. Furthermore, the sphere-forming cells expressed several CSC marker genes reversibly and were chemo-resistant, suggesting that cancer cells might be exchanged between cancer stem-like cell and differentiated cancer cell reversibly in tumors in vivo. Consistent with this idea, about 20 % of tumor cells express CD44v9 in the in vivo xenograft model either after transplantation of sphere-forming stem-like cells expressing CD44v9 or even after transplantation of monolayer cells that did not express CD44v9 (Fig. 5). Previously we found that serum starvation of *RasG12V*-transformed cell resulted in the CSC-like properties^24^. A small population of cancer cells would express CSC marker genes in vivo in consequence of adaptation to the tumor microenvironments such as low nutrition, hypoxia or low attachment.

Previously Su et al.^43^ showed that lipid raft-associated CD44 is required for survival in the suspension condition during the process of tumor metastasis, and then nuclear CD44 / acetylated-STAT3 was involved in producing cells with CSC properties and EMT phenotype by transcription reprogramming, leading to tumor initiation, tumor metastasis and drug resistance. These result suggested that CD44 expression were required for CSC-like maintenance of sphere-forming cancer cells in our experience. The leucine-rich repeat-containing G-protein-coupled receptor 5 (LGR5) indicated intestinal CSCs in murine tumors by using the mouse tumors designed to reproduce the clinical progression of human colorectal cancer^44, 45^. The authors demonstrated that selective ablation of LGR5^+^ cells restricts primary tumor proliferation, but observed tumor regrowth driven by re-expressing LGR5^+^ cancer cells after differentiation^44, 45^. These results suggested that differentiated cancer cells can continuously regenerate CSCs in tumors in the xenograft mouse model. In other words, some differentiated cancer cells potentially have an ability of conversion to CSCs. These observations are consistent with our results that differentiated cancer cells became to show CSC-like properties under serum starvation^24^ and unattached conditions (this article). Based on this idea, we should investigate the contribution of the tumor microenvironment and the position of the adaptive cell in near future.

Doxycycline treatment reduced tumor growth in pancreatic tumor xenografts and bone-associated soft-tissue tumor mass^46, 47^. However, author described that doxycycline was contributed to matrix-metalloproteinases inhibition^48^. Here, we showed that antibiotics such as doxycycline and chloramphenicol can selectively target genitourinary cancer CSCs. Mechanistically, these antibiotics converge with mitochondrial translation and OXPHOS. Thus, molecular disruption of mitochondrial OXPHOS or biogenesis can be a new target for CSC eradication in vitro and in vivo. Importantly and notably, the antibiotics did not affect the proliferation of the monolayer culture and CD44v9-negative tumors in vivo. When ER stress was induced in monolayer culture cells by thapsigargin, ER stress-induced apoptosis even in the monolayer cultures. Knockdown of ATF4 and PUMA inhibited doxycycline-induced apoptosis for the sphere-forming culture cells, suggesting that the ER stress response is a key player for antibiotics-induced apoptosis for cancer stem-like cell and a new stratagy for cancer therapy.

MAMs have been recently shown to regulate mitochondrial redox status and energy metabolism and play an important role in regulation ER stress and autophagy. These data suggested that MAM formation and mitochondrial function in cancer stem-like cell is a crucial target for chemotherapy.

Our data provide new molecular findings that cancer cells showed the CSC-like properties in a reversible manner. Translation inhibition by doxycycline affected mitochondrial function in cancer stem-like cells, subsequently promoted ER stress-induced apoptosis in vitro and suppressed tumor cell growth in vivo. Hence we consider the increase in mitochondrial respiration during the sphere formation is prerequisite for cancer stem-like cell conversion. These findings provide important insights into CSC biology and a potential new treatment of human cancers.

Finally, we propose the existence of cancer cells that can be reversibly converted to cells with CSC-like properties at least in particular species of tumors. Considering that the cancer stem-like cells are reversible and require mitochondrial respiration, a simultaneous combination of anti-cancer drugs and mitochondria-targeting antibiotics would be a potentially effective approach.

## Materials and Methods

### Cell culture

PC-3, DU145, and LNCap prostate cancer cells and RWPE-1 normal prostate cells were obtained from ATCC. Human bladder cancer KK-47, KU-7 and T24 cells were kindly provided by Dr. Osamu Ogawa (Kyoto University, Japan). All cells were authenticated in 2016 by high-resolution Small Tandem Repeat profiling (STR, GenePrint 10; Promega, Fitchburg,Wis) by BEX corporation and cultured according to each instructions. All cell lines had been subcultured for less than 16 passages and were tested to be free of mycoplasma contamination.

### Sphere-forming cell culture assay

Cells were plated in serum-free medium DMEM/F12 (Thermo Scientific) containing B27 without Vitamine A (Invitrogen, Waltham, MA), 20 ng/ml EGF and 0.4% PenStrep in non-adherent conditions, in Nunclon^TM^ sphere dishes (Thermo Scientific). The sphere forming number was counted after 3 days of culture. Sphere-forming efficiency (%) was calculated by dividing number of sphere-forming cells by the original seeded number. After 3 days, we counted sphere number which over 50 μm diameter. To change from sphere to layer, the sphere cell suspension was prepared using Accutase (12679-54) and plated in coated plates at a density of 1 × 10^5^ cells/well.

### Measurement of mitochondrial membrane potential (MMP), ROS and apoptosis by FACS

Apoptosis was measured using Annexin V-FITC/ PI apoptosis assay (Invitrogen). Layer or sphere cells were dissociated by Accutase and washed by PBS. Cells were stained with Annexin V-FITC and PI with HBSS (37°C, 10 min) and then analyzed by BD FACSVerse^TM^ (BD Biosciences). MMP and intracellular ROS value were acquired on a FACS Verse by using the specific probe tetramethyl-rhodamine-methylester and MitoSOX Red probe (Invitrogen)^24^.

### mRNA quantification and cDNA sequencing

The quantitative RT-PCR were performed as previously reported^24^. Briefly, we performed reverse transcription of 0.5 μg RNA using a PrimeScript™ RT Reagent Kit (TAKARA) and detected by using the SYBR^®^ *Premix Ex Taq*™ II (TAKARA) with a thermal cycler (StepOne plus; Applied Biosystems). CD44v8-10 genes product were detected and confirmed using the BigDye^®^ Terminator V3.1 Cycle sequencing kit (Thermo Fisher).

### Small interfering RNA (siRNA) transfection

Specific and control siRNAs were obtained and siRNA transfection at 100 nM with Lipofectamine RNAiMAX reagent were performed according to the manufacturer’s instruction (Invitrogen).^24^. Transfected cells were immediately seeded in sphere condition medium in an uncoated dish. Primers for siRNA sequence are listed in Supplementary Table S1 and S2.

### Seahorse XF24 flux Analyzer

Mitochondrial OXPHOS activity can be measured using the oxygen consumption rate (OCR) method using an XFe24 Analyzer (Seahorse Biosciences, North Billerica, MA)^24^. The monolayer or sphere-forming cells (4 × 10^4^/ well) were seeded into microplates coated with Matrigel (Corning) and then centrifuged for 2 min at 2000 rpm at 37°C. The cells were then trypsinized and counted to normalize the values.

### Immunofluorescence, immunohistochemistry and immunoblotting

Immunofluorescent staining was performed on monolayer or sphere cell cultures fixed by incubating with IPEL gel (GenoStaff, PG20-1) and sphere-forming cell were embedded in paraffin and sectioned into 2 μm thick layers, followed by staining with the specified primary antibodies (Supplemental Table S3)^24^. Relative intensities were analyzed by Imaris (BITPLANE) or Hybrid cell count BZ-H2C software (Keyence, Osaka, Japan). The immunoblotting assay were performed as described previously^49, 50^.

### Xenograft tumor formation

All xenograft analysis was performed under the guidelines of the Kyushu University Animal Research Facility and were approved by the Kyushu University Institutional Care and Use Committee under Protocol #A29-052-0. All animal experimental procedures followed Guidance for the Care and Use of Laboratory Animals, Eighth Edition, updated by the US National Research Council Committee in 2011. Monolayer and sphere PC-3 cells were re-suspended in matrigel with MEM (1:1 mixture volume), and 0.1 ml mixture was implanted subcutaneously (s.c.) into 6-week-old male Balb/c-nu mice (Charles River). Doxycycline solution (60 mg/kg/day) was administered daily to each mouse in the doxycycline treated group (4 mice) ip; a 0.9% saline solution was administered to all animals in the control normal saline-treated group (4 mice). Tumors were harvested and one-half of the tumor sample was subjected to hematoxylin-eosin or immunofluorescence staining. All experiences were done by no randomized and no blinded.

### Statistical analysis

All Student’s *t*-tests performed were two-tailed. Data were presented as mean ± SD (standard deviation). A *p*-value (*p* < 0.05 or less) was considered statistically significant.

## Electronic supplementary material

Sup S1

Sup S2

Sup S3

Sup S4

Sup S5

Sup S6

Supplementary Tables
