# Supplementary material for: Doxycycline induces apoptosis via ER stress selectively to cells with a cancer stem cell-like properties: importance of stem cell plasticity
Source: Oncogenesis. 2017 Nov 29;6(11):397. doi: 10.1038/s41389-017-0009-3 (PMC5868058; doi:10.1038/s41389-017-0009-3)
Supplement: Supplementary file 1 — Sup S1 [file 41389_2017_9_MOESM1_ESM.pdf]

Supplementary Figure S1

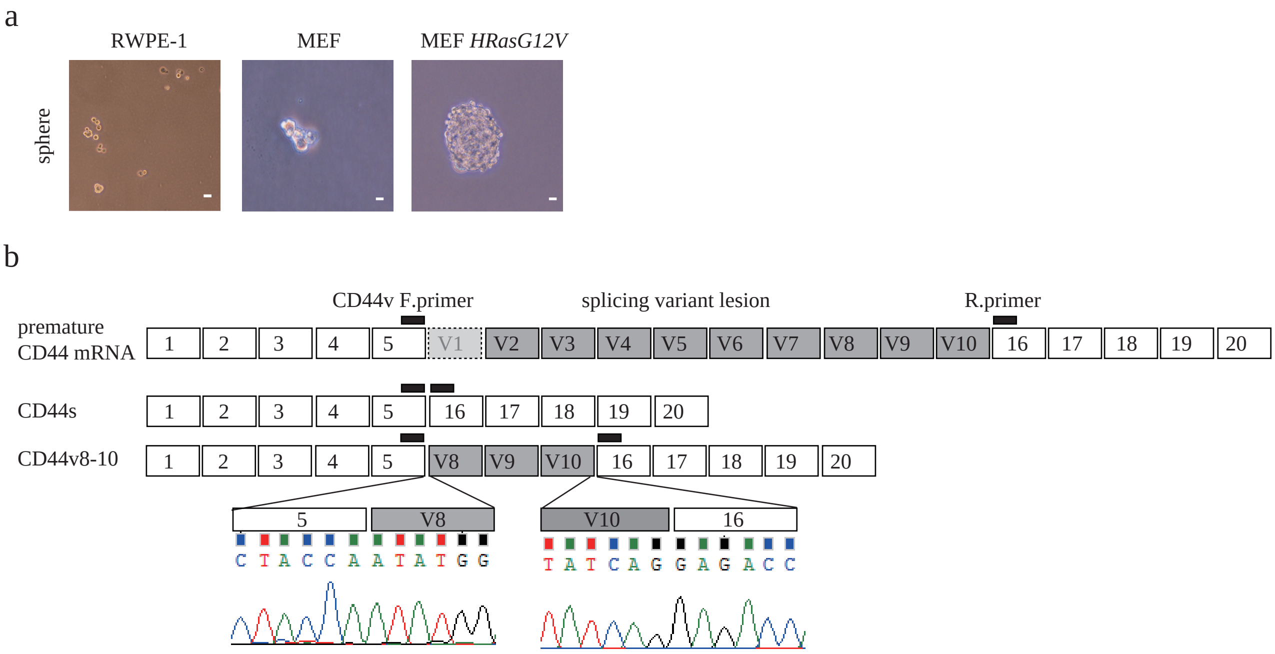

>CD44v8-10 direct sequence matches NM_001001390

1078 gcacagacagaatccctgctaccaatatggactccagtcatagtataacgcttcagcctactgcaaatccaaacacaggtttggtggaagatttggacaggacaggacctctttcaatgacaacgcag

cagagtaattctcagagcttctctacatcacatgaaggcttggaagaagataaagaccatccaacaacttctactctgacatcaagcaataggaatgatgtcacaggtggaagaagagacccaaatcattctga

aggctcaactactttactggaaggttatacctctcattacccacacacgaaggaaagcaggaccttcatcccagtgacctcagctaagactgggtcctttggagttactgcagttactgttggagattccaactcta

atgtcaatcgttccttatcaggagaccaagacacattccaccccagtggggggtcccataccactcatggatctgaatcagatggacactcacatgggagtcaagaaggtggagca 1592

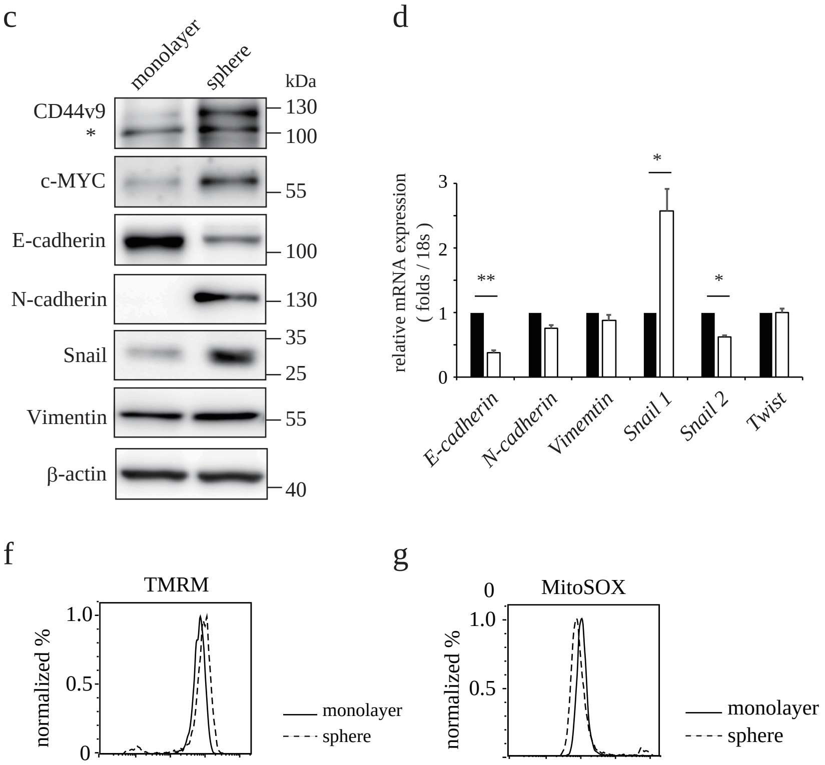

Supplementary Legend S1

Oncogenesis and the mitochondrial electron transport chain are required for sphere formation, which indicates CD44v8-10 expression and epithelial to mesenchymal transition.

(**a**) Bright-field image in sphere-forming RWPE-1, MEF and *RasG12V* transformed MEF cells. Scale bar = 10μm. (**b**) Schema indicates CD44 variant mRNA. CD44v1 is silent in humans because of containing a stop codon in rats and mice. The entire variant region is amplified with CD44v primers such as the schema. Direct sequencing matches NM_001001390 (CD44v8-10) and shows the exon gap between exon5 and v8, v10 and exon16. (**c**) Immunoblotting analysis of CD44v9, c-MYC, E-cadherin, N-cadherin, Snail, and Vimentin protein in monolayer and sphere-forming PC-3 cells. β-actin was used as an internal control. The asterisk indicates a non-specific band. (**d**) Relative mRNA expression of EMT marker in monolayer and sphere-forming PC-3 cells. Data were normalized to the expression level in monolayer cells for each RNA species. Data shows the mean ± SD of triplicates. $*p < 0.05$, $**p < 0.01$. (**e**) Bright-field image in sphere-forming osteosarcoma 143B and Rho^0 cells. The lower panel shows relative cell number of spheres in 143B and Rho^0 cells. Data were normalized to the cell number in 143B. (**f**, **g**) Flow cytometric analysis of TMRM and mitoSOX in monolayer and sphere-forming PC-3 cells.
