# Supplementary material for: Doxycycline induces apoptosis via ER stress selectively to cells with a cancer stem cell-like properties: importance of stem cell plasticity
Source: Oncogenesis. 2017 Nov 29;6(11):397. doi: 10.1038/s41389-017-0009-3 (PMC5868058; doi:10.1038/s41389-017-0009-3)
Supplement: Supplementary file 2 — Sup S2 [file 41389_2017_9_MOESM2_ESM.pdf]

Supplementary Figure S2

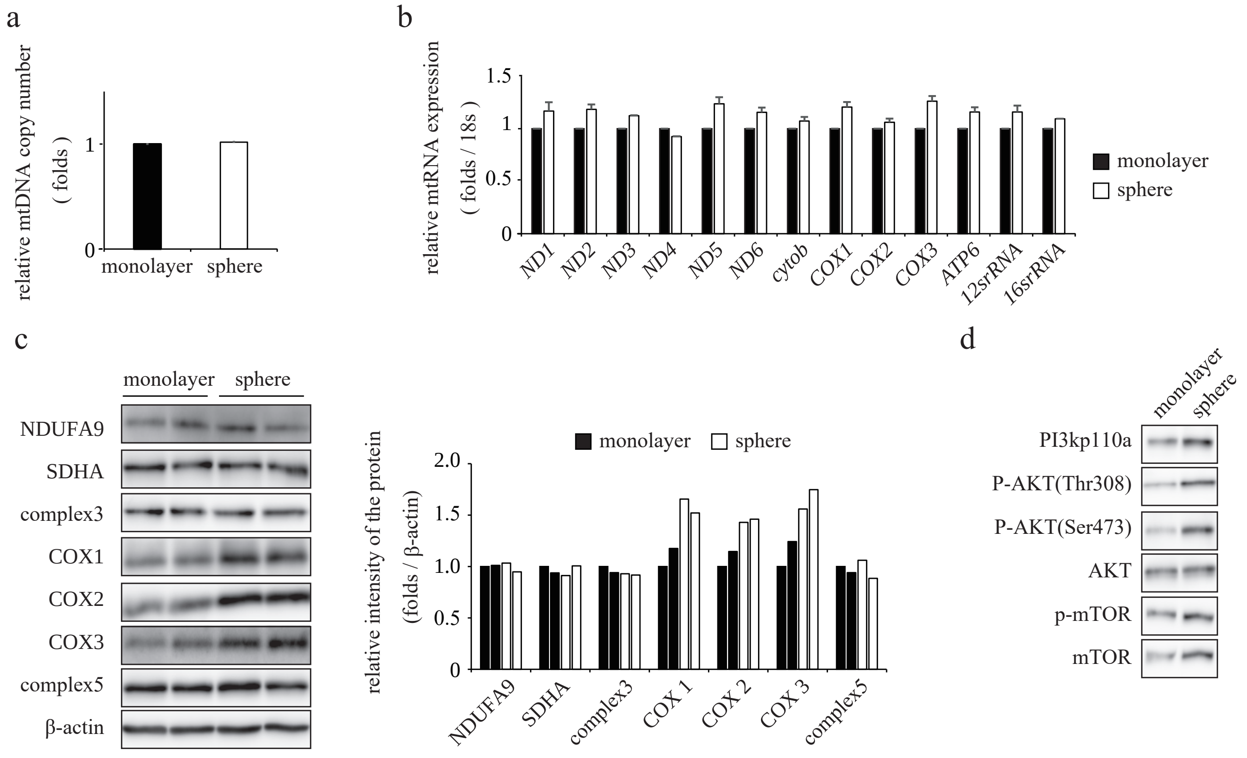

Supplementary Legend S2

Mitochondrial translation, not DNA copy number and gene expression, is increased and PI3K-AKT pathway is activated in sphere-forming cells.

(a) Relative mtDNA copy number in monolayer and sphere-forming PC-3 cells. Data were normalized to the mtDNA level in monolayer. (b) Relative mtRNA expression in monolayer and sphere-forming PC-3 cells. Data were normalized to the expression level in monolayer cells for each RNA species. Data shows the mean ± SD of triplicates. (c) Immunoblotting analysis of NDUFA9, SDHA, complex3, COX1, COX2, COX3, complex5 and β-actin protein in monolayer and sphere-forming PC-3 cells. In the right panel, relative intensity of the proteins were shown. Data were normalized to the intensity level in monolayer. (d) Immunoblotting analysis of PI3kp110α, P-AKT (Thr308), P-AKT (Ser473), AKT, p-mTOR and mTOR in monolayer and sphere-forming PC-3 cells.
