# Supplementary material for: Doxycycline induces apoptosis via ER stress selectively to cells with a cancer stem cell-like properties: importance of stem cell plasticity
Source: Oncogenesis. 2017 Nov 29;6(11):397. doi: 10.1038/s41389-017-0009-3 (PMC5868058; doi:10.1038/s41389-017-0009-3)
Supplement: Supplementary file 3 — Sup S3 [file 41389_2017_9_MOESM3_ESM.pdf]

Supplementary Figure S3

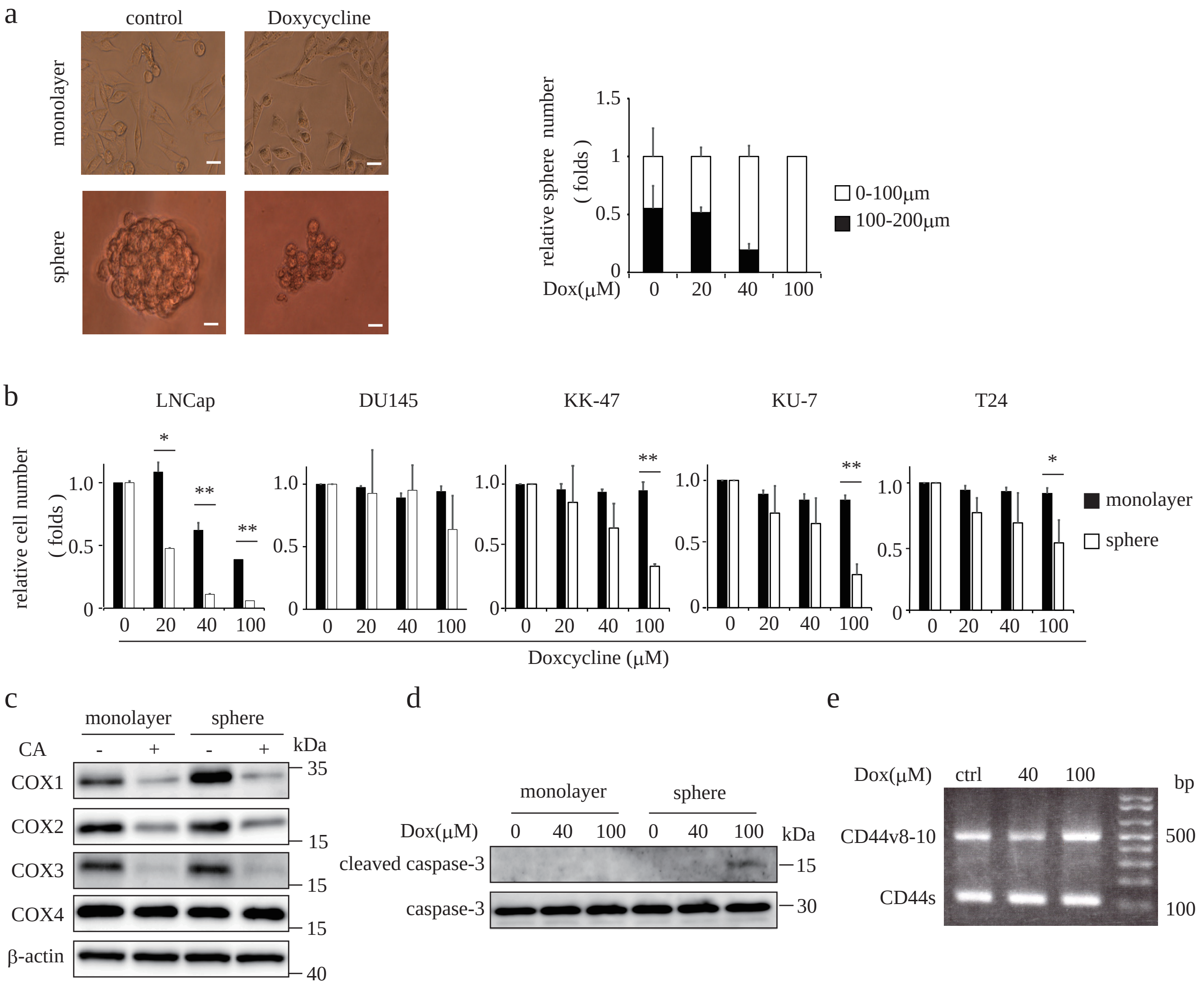

Supplementary Legend S3

Doxycycline inhibits the growth in the various sphere-forming cancer cells.

(a) Bright-field image in monolayer and sphere-forming PC-3 cells with 40μM doxycycline for 24h. Scale bar = 10μm. In the right panel, relative sphere number treated with various concentrations of doxycycline (Dox) were shown. Data were normalized to the total sphere number. Data shows the mean ± SD of triplicates. (b) Relative cell number compared to no drug treatment by MTS assay in monolayer and sphere-forming cells of LNCap, DU145, KK-47, KU-7 and T24 treated with various concentrations of doxycycline. Data shows the mean ± SD of triplicates. *p < 0.05, **p < 0.01. (c) Immunoblotting analysis of COX1, COX2, COX3, COX4 and β-actin protein in monolayer and sphere-forming PC-3 cells treated with 100μM Chloramphenicole (CA). (d) Immunoblotting analysis of caspase-3 and cleaved caspase-3 in monolayer and sphere-forming PC-3 cells treated with various concentrations of Dox. (e) CD44v8-10 expression in sphere-forming PC-3 cells after doxycycline treatment.
