# Supplementary material for: Doxycycline induces apoptosis via ER stress selectively to cells with a cancer stem cell-like properties: importance of stem cell plasticity
Source: Oncogenesis. 2017 Nov 29;6(11):397. doi: 10.1038/s41389-017-0009-3 (PMC5868058; doi:10.1038/s41389-017-0009-3)
Supplement: Supplementary file 4 — Sup S4 [file 41389_2017_9_MOESM4_ESM.pdf]

Supplementary Figure S4

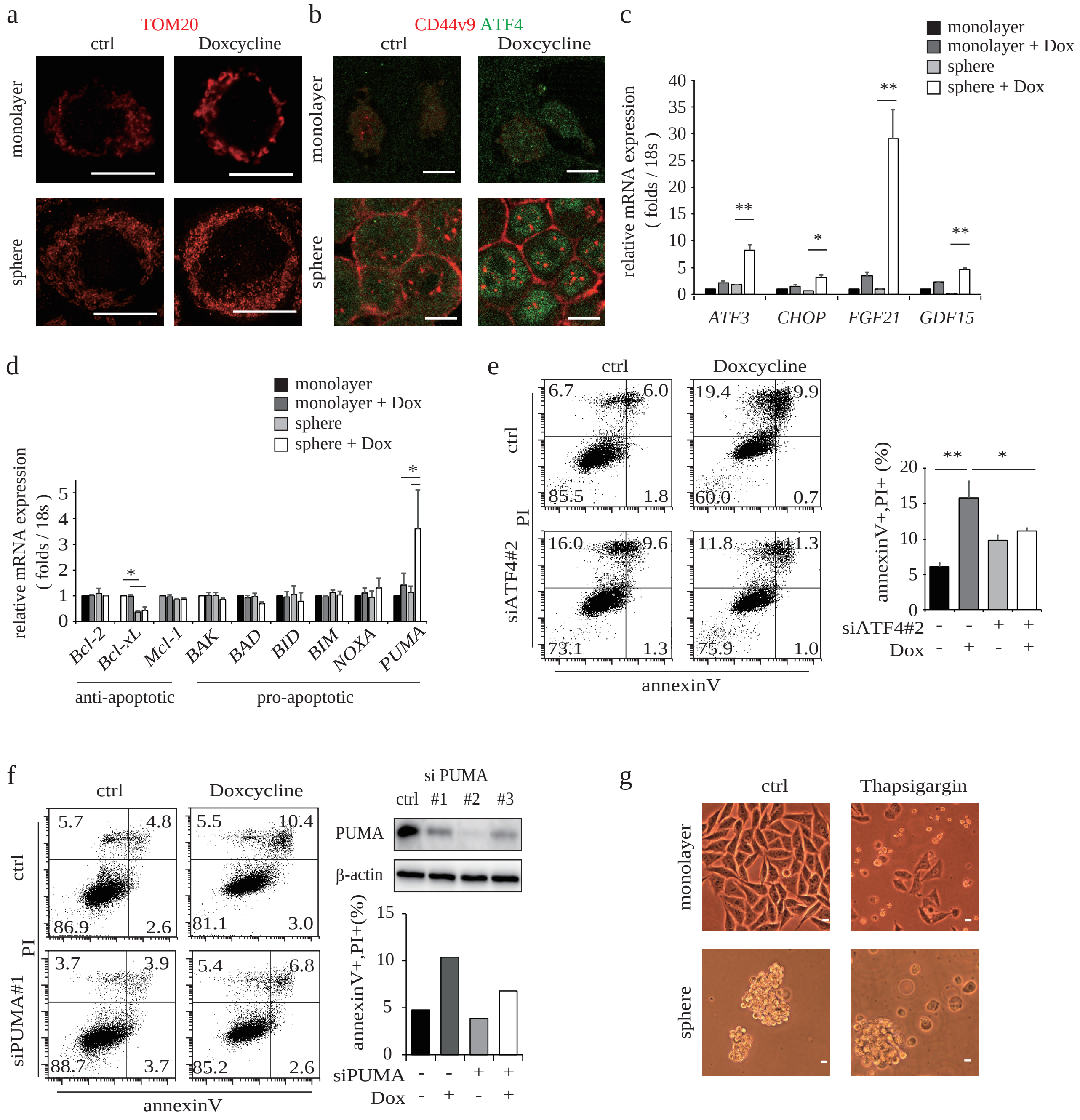

Supplementary Legend S4

ER stress and pro-apoptotic PUMA expression contribute to the apoptosis in sphere-forming cells.

(**a**) Immunofluorescence staining of TOM20 in monolayer and sphere-forming PC-3 cells treated with 100μM doxycycline for 24h. Scale bar = 10μm. (**b**) Immunofluorescence staining of CD44v9 and ATF4 in monolayer and sphere-forming PC-3 cells treated with 40μM doxycycline. Scale bar = 10μm. (c) Relative mRNA expression of ATF3, CHOP, FGF21, GDF15 in monolayer and sphere-forming PC-3 cells treated with 40 μM doxycycline (Dox). Data were normalized to the expression level in monolayer for each RNA species. Data shows the mean ± SD of triplicates. *$p < 0.05$, **$p < 0.01$. (d)Relative mRNA expression of anti-apoptotic Bcl2 family (Bcl2, Bcl-xL, Mcl-1) and pro-apoptotic Bcl-2 family (BAK, BAD, BID, BIM, NOXA, PUMA) in monolayer and sphere-forming PC-3 cells treated with 40μM Dox. Data were normalized to the expression level in monolayer for each RNA species. Data shows the mean ± SD of triplicates. *$p < 0.05$. (**e**) Flow cytometric analysis of apoptosis in sphere-forming cells transfected with ATF4#2 siRNA and treated with 40 μM doxycycline for 24 h. In the right panel, the rates of subpopulations of Annexin V (+)/PI (+) are shown. *$p < 0.05$, **$p < 0.01$. (f) Flow cytometric analysis of apoptosis in spheres transfected with PUMA#1 siRNA treated with 40 μM doxycycline for 24 h. In the right panel, the subpopulations of Annexin V (+)/PI (+) are shown. Immunoblotting analysis of PUMA in siRNA-transfected cells confirm siRNA efficacy. β-actin protein serves as an internal loading control. (g) Bright-field image in monolayer and sphere-forming PC-3 cells treated with 1μM Thapsigargin. Scale bar = 10μm.
