# Supplementary material for: Doxycycline induces apoptosis via ER stress selectively to cells with a cancer stem cell-like properties: importance of stem cell plasticity
Source: Oncogenesis. 2017 Nov 29;6(11):397. doi: 10.1038/s41389-017-0009-3 (PMC5868058; doi:10.1038/s41389-017-0009-3)
Supplement: Supplementary file 5 — Sup S5 [file 41389_2017_9_MOESM5_ESM.pdf]

Supplementary Figure S5

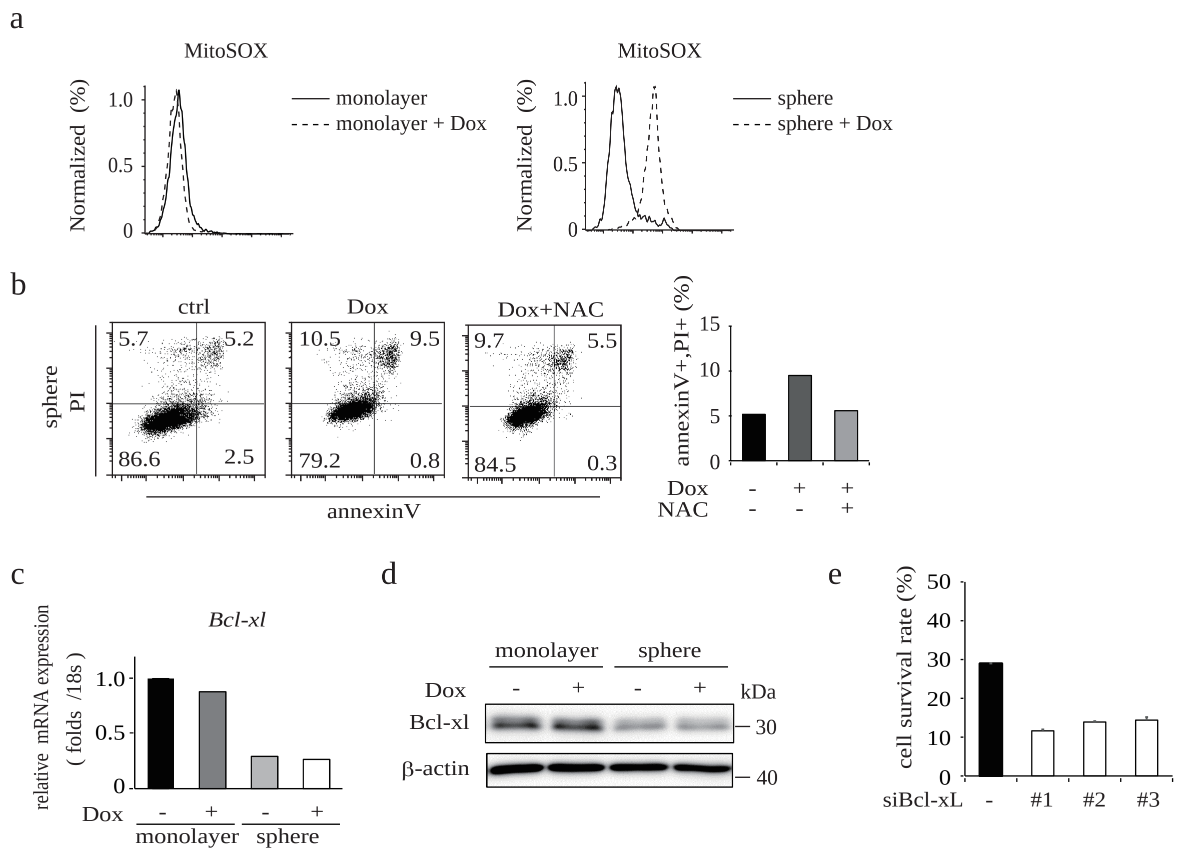

Supplementary Legend S5

ROS production and anti-apoptotic Bcl-xL reduction is also involved in doxycycline-induced apoptosis in sphere-forming cells.

(a) Flow cytometric analysis of MitoSOX in monolayer and sphere-forming PC-3 cells treated with 100 μM doxycycline for 24h. (b) Flow cytometric analysis of apoptosis in sphere-forming PC-3 cells treated with 40 μM doxycycline (Dox) and N-acetyl-cysteine (NAC). In the right panel, the rates of subpopulation of AnnexinV(+)/PI(+) are shown. (c) Relative mRNA expression of Bcl-xL in monolayer and sphere-forming PC-3 cells treated with 40μM doxycycline. Data were normalized to the expression level in monolayer. (d) Immunoblotting analysis of Bcl-xL protein in monolayer and sphere-forming PC-3 cells treated with 40μM Dox. β-actin was used as an internal control. (e) Cell survival rate by MTS assay in sphere forming PC-3 cells transfected with Bcl-xL#1, #2, #3 siRNA and treated with 100 μM doxycycline for 24 h.
