# Supplementary material for: Doxycycline induces apoptosis via ER stress selectively to cells with a cancer stem cell-like properties: importance of stem cell plasticity
Source: Oncogenesis. 2017 Nov 29;6(11):397. doi: 10.1038/s41389-017-0009-3 (PMC5868058; doi:10.1038/s41389-017-0009-3)
Supplement: Supplementary file 6 — Sup S6 [file 41389_2017_9_MOESM6_ESM.pdf]

Supplementary Figure S6

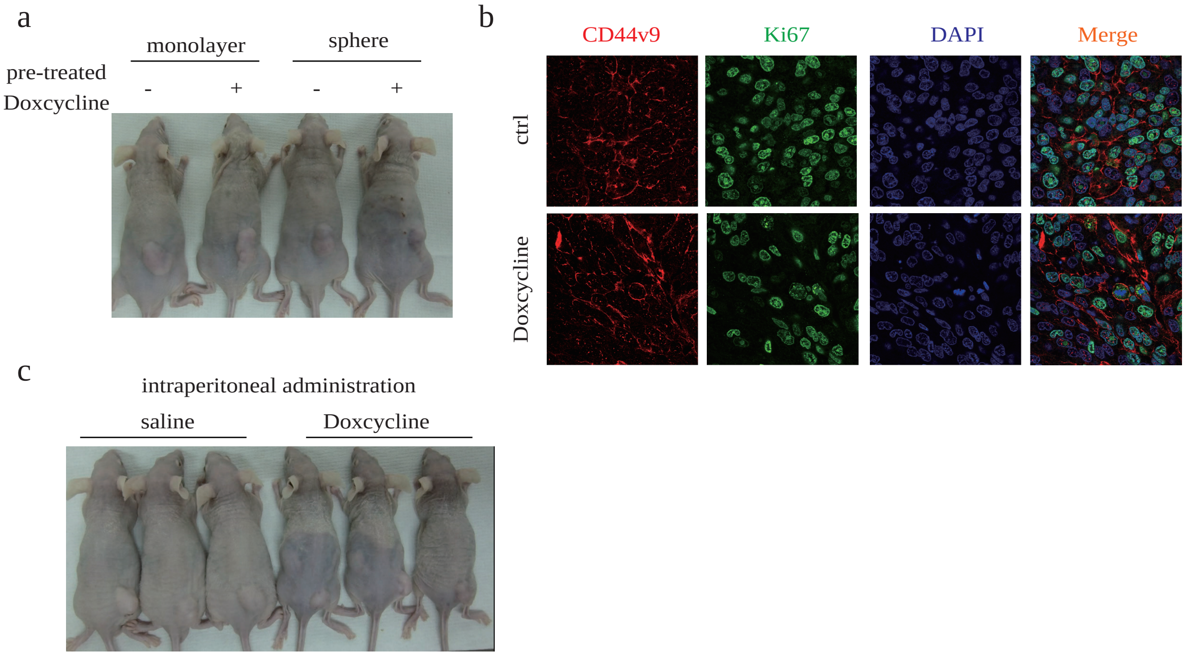

Supplementary Legend S6

Pre-treated and intraperitoneal Doxycycline administration inhibit the tumor growth in a xenograft model.

(a) Tumor volume in Balb/c-nu at 19 days after the xenografts were pre-treated with 40 μM doxycycline (Dox) n = 3 for each group. (b) Immunofluorescence staining of CD44v9 Ki67 and DAPI in the xenograft model treated with saline or doxycycline. Scale bar = 10 μm. (c) Changes in tumor volume in Balb/c-nu at 15 days after the xenograft. n = 4 for each group. The mice were treated with 60 mg/kg doxycycline or 0.9 % saline by intraperitoneal administration for 15 consecutive days after sphere-forming cells were implanted.
