# Supplementary material for: Doxycycline induces apoptosis via ER stress selectively to cells with a cancer stem cell-like properties: importance of stem cell plasticity
Source: Oncogenesis. 2017 Nov 29;6(11):397. doi: 10.1038/s41389-017-0009-3 (PMC5868058; doi:10.1038/s41389-017-0009-3)
Supplement: Supplementary file 7 — Supplementary Tables [file 41389_2017_9_MOESM7_ESM.pdf]

Supplementary Material

Supplementary Table 1

Primer sequences of the RT-PCR primers are as follows

Primer	Forward (5’ - 3’)	Reverse (5’ - 3’)
SOX2	agaaccccaagatgcacaac	cgtctccgacaaaagtttcc
c-MYC	tgctccatgaggagacacc	gatccagactctgaccttttgc
OCT4	cgaaagagaaagcgaaccag	gccggttacagaaccacact
KLF4	tcccatctttctccacgttc	agtcgcttcatgtgggagag
ALDH1A1	gaaagagcccttgcattgtg	tggatgcggctatacaacac
CD44	ggctttcaatagcaccttgc	gttgtttgctgcacagatgg
BCL2	ttccgcgtgattgaagacac	tcccagaggaaaagcaacgg
Bcl-xL	acgagtttgaactgcggtac	gctgctgcattgttcccatag
MCL1	aatgtgctgctggcttttgc	aactggttttggtggtggtg
BAK1	agttccagaccatgttgcag	ccccaattgatgccactctc
BAX	tctgacggcaacttcaactg	agtccaatgtccagcccatg
BAD	tgagtgacgagtttgtggactc	ccaagttccgatcccaccag
BID	tgctccgtgatgtctttcac	agtcacacttctggaactgtcc
BIM	atcatcgcggtattcggttc	ggttgctttgccatttggtc
NOXA	gaagaaggcgcgcaagaac	tgccggaagttcagtttgtc
PUMA	gagatggagcccaattaggtg	tccagtatgctacatggtgcag
ATF3	ggtttgccatccagaacaag	cgtcgcctctttttcctttc
ATF4	tcaaacctcatgggttctcc	tctccaacatccaatctgtcc
CHOP	tggtatgaggacctgcaagag	agtcagccaagccagagaag
ND1	atggccaacctcctactcct	gcggtgatgtagagggtgat
ND2	aagcaaccgcatccataatc	tcagaagtgaaagggggcta
ND3	accacaactcaacggctaca	ttgtagggctcatggtaggg
ND4	cctgactcctacccctcaca	atcgggtgatgatagccaag
ND5	acatctgtacccacgccttc	tcgatgatgtggtctttgga
ND6	tgattgttagcggtgtggtc	ccacagcaccaatcctacct
cytB	tatccgccatcccatacatt	ggtgattcctagggggttgt
COX1	ggcctgactggcattgtatt	tggcgtaggtttggtctagg
COX2	ttcatgatcacgccctcata	taaaggatgcgtagggatgg
COX3	cccgctaaatcccctagaag	ggaagcctgtggctacaaaa
ATP6	tattgatccccacctccaaa	gatggccatggctaggttta
12srRNA	aaactgctcgccagaacact	catgggctacaccttgacct

16srRNA	gctaaacctagccccaaacc	ttggctctccttgcaaagtt
CD44v	gcacagacagaatccctgct	gctccaccttcttgactccc
E-cadherin	ttgaacgaatggggcaatcg	accagcaacgtgatttctgc
N-cadharin	aaacagcaacgacgggttag	aacagacacggttgcagttg
Vimentin	tctcagcatcacgatgaccttg	ttgcgctcctgaaaaactgc
Snail1	ttcccatggccatttctgtg	acaaaaacccacgcagacag
Snail2	acgcctccaaaaagccaaac	acacagtgatggggctgtatg
Twist1	ataagagcctccaagtctgcag	aaaaagaaagcgcccaacgg

Supplementary Table 2

Target sequences of siRNA are as follows

Gene	5'-3' target sequences of siRNA	Company
si ATF4 #1	GCCUUCUCCGGGACAGAUUtt	genosys
si ATF4 #2	GCUCUUACUGGUGAGUGCAtt	genosys
si ATF4 #3	GAUAGGAAGCCAGACUACAtt	genosys
si PUMA #1	GGCGGAGACAAGAGGAGCAtt	GeneDesign,Inc
si PUMA #2	GAGCCAAACGUGACCACUAtt	GeneDesign,Inc
si PUMA #3	GGUGGGAGAUUUUGGCUGAtt	GeneDesign,Inc
si Bcl-xL #1	GGAUACAGCUGGAGUCAGUtt	GeneDesign,Inc
si Bcl-xL #2	CCAGGGAGCUUGAAAGUUUtt	GeneDesign,Inc
si Bcl-xL #3	CCACUUUUGACUAGGGAUUtt	GeneDesign,Inc

Supplementary Table 3

Primary antibodies are as follows

Primary antibody	number	company
CD44v9	LKG-M003	COSMO BIO
Parkin	ab77924	Abcam
PINK1	ab75487	Abcam
NDUFA9	ab14713	Abcam
SDHA	ab14715	Abcam
Complex3(Ubiquinol-Cytochrome C Reductase Core Protein I)	ab110252	Abcam
COX1(MTCO1)	ab14705	Abcam
COX2(MTCO2)	ab79393	Abcam
COX3(MTCO3)	ab110259	Abcam

ATP5A	ab14748	Abcam
FACL4	ab137525	Abcam
PUMA	ab33906	Abcam
Drp1	611112	BD
OPA1	615606	BD
E-cadherin	#3195P	CST
BCLXL	#2764s	CST
cleaved caspase-3	#9664P	CST
caspase-3	#9665	CST
Mfn2(mitofusin-2)	#9482	CST
PI3kp110α	#4249S	CST
P-AKT(Thr308)	#2965S	CST
P-AKT(Ser473)	#4060	CST
AKT	#4691P	CST
p-mTOR	#2971	CST
mTOR	#2983P	CST
COX4	PM063	MBL life science
c-MYC	Sc-40	Santa Cruz Biotechnology
α-tubulin	sc-5286	Santa Cruz Biotechnology
TOM20(Rb)	sc-11415	Santa Cruz Biotechnology
TOM20(Mo)	sc-17764	Santa Cruz Biotechnology
β-actin	A5441	Sigma Aldrich
N-cadherin	610920	BD
Vimentin	550513	BD
Snail	#3879	CST
anti-mouse IgG HRP-linked	#7076S	CST
anti-rabbit IgG HRP-linked	#7074	CST
anti-rat IgG HRP-linked	#7077S	CST
Alexa Fluor® 488 F(ab')2 Fragment of Goat Anti-Rabbit IgG (H+L)	#A11070	Invitrogen
Alexa Fluor® 594 F(ab')2 Fragment of Goat Anti-mouse IgG (H+L)	#A11080	Invitrogen
Alexa Fluor® 594 F(ab')2 Fragment of Goat Anti-Rat IgG (H+L)	#A11007	Invitrogen
